# Measurement of overall insecticidal effects in experimental hut trials

**DOI:** 10.1186/1756-3305-5-256

**Published:** 2012-11-13

**Authors:** Olivier JT Briët, Thomas A Smith, Nakul Chitnis

**Affiliations:** 1 Department of Epidemiology and Public Health, Swiss Tropical and Public Health Institute, Socinstrasse 57, 4051, Basel, Switzerland; 2 University of Basel, Petersplatz 1, 4003, Basel, Switzerland

**Keywords:** Experimental hut, Insecticidal effect, Effectiveness, Mosquitoes, Vectors, Malaria, Insecticides, Protection

## Abstract

**Background:**

The ‘overall insecticidal effect’ is a key measure used to evaluate public health pesticides for indoor use in experimental hut trials. It depends on the proportion of mosquitoes that are killed out of those that enter the treated hut, intrinsic mortality in the control hut, and the ratio of mosquitoes entering the treatment hut to those entering the control hut. This paper critically examines the way the effect is defined, and discusses how it can be used to infer effectiveness of intervention programmes.

**Findings:**

The overall insecticidal effect, as defined by the World Health Organization in 2006, can be negative when deterrence from entering the treated hut is high, even if all mosquitoes that enter are killed, wrongly suggesting that the insecticide enhances mosquito survival. Also in the absence of deterrence, even if the insecticide kills all mosquitoes in the treatment hut, the insecticidal effect is less than 100%, unless intrinsic mortality is nil. A proposed alternative definition for the measurement of the overall insecticidal effect has the desirable range of 0 to 1 (100%), provided mortality among non-repelled mosquitoes in the treated hut is less than the corresponding mortality in the control hut. This definition can be built upon to formulate the coverage-dependent insecticidal effectiveness of an intervention programme. Coverage-dependent population protection against feeding can be formulated similarly.

**Conclusions:**

This paper shows that the 2006 recommended quantity for measuring the overall insecticidal effect is problematic, and proposes an alternative quantity with more desirable properties.

## Findings

### Background

Phase II experimental hut trials are the main means of evaluating insecticides used in indoor interventions against mosquitoes at field level. The World Health Organization Pesticide Evaluation Scheme (WHOPES) provides guidelines on how such trials should be carried out and analysed [1]. The potential impact of insecticides on disease transmission is measured by two key effects: personal protection against bites, and the overall insecticidal effect. The overall insecticidal effect depends on the killing of mosquitoes that enter the hut and the deterrence of mosquitoes from entering the hut, compared to a hut without the intervention.

### Overall insecticidal effect in an experimental hut study

The data collected from an experimental hut (with some intervention treatment) generally include counts^a^ of the number of female mosquitoes entering, *E*_*t*_, and of those, the number dead, *D*_*t*_, in a defined period. Sometimes, also the number of unfed live females caught, *U*_*t*_ is recorded. Corresponding to these counts are the numbers entering, *E*_0_, unfed alive, *U*_0_, and those dead, *D*_0_, in negative control huts (without insecticide) matched in space and time. Of those mosquitoes that enter the treated hut, the proportion that die is then

(1) $$\mu_{t}=\frac{D_{t}}{E_{t}}=\mu_{t}^{⋆}\left(1-\rho_{t}\right),$$

where *ρ*_*t*_ = *U*_*t*_/*E*_*t*_ is the proportion of females repelled^b^ after entry, and *μ*_*t*_^⋆^ = *D*_*t*_/(*E*_*t*_ − *U*_*t*_) is the proportion of dead mosquitoes out of those that were not repelled after entry. Similarly, among those that enter the control hut, the intrinsic mortality is estimated by

(2) $$\mu_{0}=\frac{D_{0}}{E_{0}}=\mu_{0}^{⋆}\left(1-\rho_{0}\right).$$

Using Abbott’s formula for “per cent control” [2], the proportion of mosquitoes that enter the treated huts that are killed by the insecticide is then the excess risk of being killed, *μ*_*t*_ − *μ*_0_. The ‘proportion control’ or efficacy, *ω*, of the intervention to kill these mosquitoes is obtained by dividing the excess risk by the proportion of mosquitoes entering the hut that would have survived, if not for the intervention treatment, i.e.

(3) $$\omega =\frac{\mu_{t}-\mu_{0}}{1-\mu_{0}}=\frac{\frac{E_{0}}{E_{t}}D_{t}-D_{0}}{E_{0}-D_{0}}.$$

The ‘proportion control’ *ω*, which can also be called ‘control-corrected mortality’, measures the killing effect of the treatment in cases where there is no effect on hut entry. However, if the intervention contains insecticides, these are expected to deter^c^ some mosquitoes from entering the hut. While deterrence benefits the occupants, whose exposure to bites is reduced, it reduces the overall effect on mosquito survival, since these deterred mosquitoes can find hosts in untreated huts without exposing themselves to the insecticide. The deterrence, *δ*, defined as the proportionate reduction in the entry rate, is then

(4) $$\delta =1-\frac{E_{t}}{E_{0}}$$

assuming that the entry rate in treated huts is less than or equal to the entry rate in untreated huts, so 0 ≤ *δ* ≤ 1.

To include the effects of deterrence in the control-corrected mortality, *ω*, the “overall insecticidal effect” [3] has been calculated as

(5) $$\sigma =\frac{\mu_{t}\left(1-\delta \right)-\mu_{0}}{1-\mu_{0}}=\frac{D_{t}-D_{0}}{E_{0}-D_{0}}.$$

WHOPES [1] recommends that, instead of measuring the overall insecticidal effect as the proportion of host seeking mosquitoes that would have survived in a control hut, it should be measured as the difference between treated and control huts in the number of mosquitoes found dead, expressed as a proportion of those host seeking, i.e.

(6) $$\sigma^{'}=\frac{D_{t}-D_{0}}{E_{0}}=\mu_{t}\left(1-\delta \right)-\mu_{0}=\sigma \left(1-\mu_{0}\right).$$

The definition *σ* seems preferable to *σ*^′^ as an ‘overall insecticidal effect’ measure because the former corrects for the mosquitoes that would have died anyway in the absence of insecticide. The value of the latter, *σ*^′^, is lower with increasing *μ*_0_, at constant *μ*_*t*_; with *μ*_0_ > 0, *σ*^′^ can never be 1, even if *μ*_*t*_ = 1. If *δ* is large, more dead mosquitoes may be found in control huts than in those treated, rendering both *σ* and *σ*^′^ negative, even if *μ*_*t*_  ≥  *μ*_0_, wrongly suggesting that the intervention enhances mosquito survival. For instance, if *μ*_*t*_ = 1, *μ*_0_ = 0.3, and *δ* = 0.8, then *σ* = − 0.143 and *σ*^′^ = − 0.1, which are counter intuitive values, especially given that the control-corrected mortality *ω* = 1.

The deterred and repelled mosquitoes must seek blood meals at least once per feeding cycle and, in a ‘natural’ situation without traps, if treated huts are rare, the worst-case scenario should be that they are diverted to huts comparable with the controls and die with probability^d^*μ*_0_^⋆^. This assumption can be captured by observing that the mean mortality of mosquitoes approaching a treated hut should be the weighted average

(7) $${\bar{\mu }}^{⋆}=\left(1-\delta \right)\left(1-\rho_{t}\right)\mu_{t}^{⋆}+\left[1-\left(1-\delta \right)\left(1-\rho_{t}\right)\right]\mu_{0}^{⋆}.$$

Note that ${\bar{\mu }}^{⋆}$ can be biased if a proportion of the mosquitoes that enter huts is exophagic. Using Abbott’s logic (mosquitoes that would die anyway do not contribute to the insecticidal effect), the overall insecticidal effect of a treated hut is

(8) $$\text{Ψ}=\frac{{\bar{\mu }}^{⋆}-\mu_{0}^{⋆}}{1-\mu_{0}^{⋆}}=\left(\frac{\mu_{t}^{⋆}-\mu_{0}^{⋆}}{1-\mu_{0}^{⋆}}\right)\left(1-\delta \right)\left(1-\rho_{t}\right).$$

**Figure 1:**
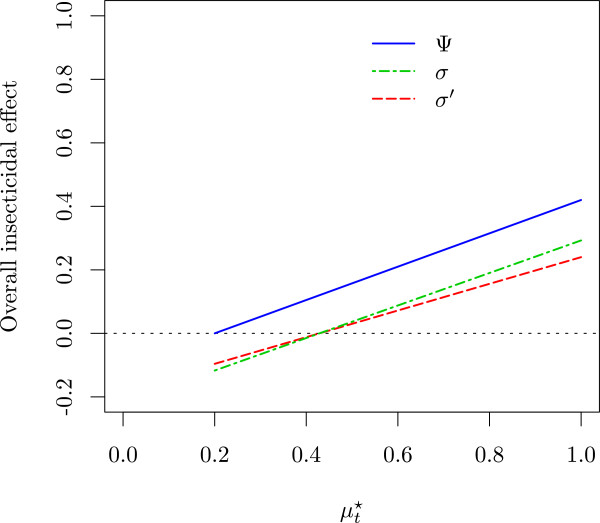
**Overall insecticidal effect quantities depending on mortality in an experimental hut with an intervention.** The proportion deterred from entering *δ* is kept constant at 0.4, and the proportion dead out of those that entered into an untreated control hut *μ*_0_^⋆^ is kept constant at 0.20. Also, the proportion repelled (unfed alive mosquitoes) out of those entered is kept constant at *ρ*_*t*_ = 0.3 and *ρ*_*0*_ = 0.1 for treated and control huts, respectively. *μ*_*t*_^⋆^ is the proportion of mosquitoes dead in an experimental hut out of those that entered. Lines are drawn for *μ*_*t*_^⋆^ ≥ *μ*_0_^⋆^.

In Figure 1, the three quantities for overall insecticidal effect Ψ, *σ* and *σ*^′^ are shown depending on mortality in huts with intervention treatment, *μ*_t_^⋆^, with a fixed mortality in control huts, *μ*_0_^⋆^, a fixed deterrence, *δ*, and fixed repellence, *ρ*_*t*_ and *ρ*_0_.

The graph of Ψ is a straight line passing through 0 at *μ*_*t*_^⋆^ = *μ*_0_^⋆^, and through (1 − *δ*)(1 − *ρ*_*t*_) at *μ*_*t*_^⋆^ = 1 with slope (1 − *δ*)(1 − *ρ*_*t*_)/(1 − *μ*_0_^⋆^), and Ψ is positive for *μ*_*t*_^⋆^ > *μ*_0_^⋆^. *σ* runs parallel to Ψ, and is negative for $\mu_{t}^{⋆}<\frac{1-\rho_{0}}{\left(1-\delta \right)\left(1-\rho_{t}\right)}\mu_{0}^{⋆}$. *σ*^′^ is also negative for $\mu_{t}^{⋆}<\frac{1-\rho_{0}}{\left(1-\delta \right)\left(1-\rho_{t}\right)}\mu_{0}^{⋆}$, but has smaller slope (1 − *δ*)(1 − *ρ*_*t*_).

### Insecticidal effectiveness in intervention programmes

**Figure 2:**
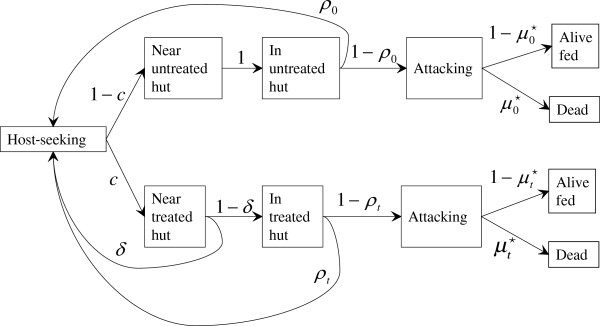
**Mosquito host seeking branching process.** Mosquito states are represented by rectangular boxes, connected by arrows representing transitions between states, with the transition probabilities in unboxed text next to the arrows.

In programmatic application, the population level insecticidal effectiveness depends on, in addition to the overall insecticidal effect, the coverage, *c*, since mosquitoes diverted from a treated hut may encounter another treated hut, with probability *c*, so that the insecticidal effectiveness increases with coverage. Assuming no shifts to outdoor biting and resting, no additional mortality while host-seeking, and assuming treated and untreated huts to be perfectly mixed, the mosquitoes’ trajectory follows that shown in Figure 2, which allows deterred and repelled mosquitoes to repeatedly approach huts until blood fed (or dead). The first time that mosquitoes approach, a proportion *t* = *c*(1 − *δ*)(1 − *ρ*_*t*_) ends up in treated huts, and a proportion *u* = (1 − *c*)(1 − *ρ*_0_) ends up in untreated huts. The remainder, *r* = 1 − [*c*(1 − *δ*)(1 − *ρ*_*t*_) + (1 − *c*)(1 − *ρ*_0_)], either deterred or repelled the first time, approach again, and of these mosquitoes, again a proportion *t* ends up in treated huts and a proportion *u* ends up in untreated huts. The total ending up in treated huts is the infinite sum *t* + *rt* + *r*^2^*t* + … = *t*/(1 − *r*), and the total ending up in untreated huts is the infinite sum *u* + *ru* + *r*^2^*u* + … = *u*/(1 − *r*). The mean mosquito mortality is then

(9) $${\bar{\mu }}_{c}^{⋆}=\frac{c\left(1-\delta \right)\left(1-\rho_{t}\right)\mu_{t}^{⋆}+\left(1-c\right)\left(1-\rho_{0}\right)\mu_{0}^{⋆}}{c\left(1-\delta \right)\left(1-\rho_{t}\right)+\left(1-c\right)\left(1-\rho_{0}\right)}.$$

Note that if *c* = 1, then ${\bar{\mu }}_{c}^{⋆}=\mu_{t}^{⋆}$ and if c = 0, then ${\bar{\mu }}_{c}^{⋆}=\mu_{0}^{⋆}$. The population-level insecticidal effectiveness, depending on *c*, is the mean mortality with intervention, compared to the situation without intervention, thus with *c* = 0:

(10) $${\text{Ψ}}_{c}=\frac{{\bar{\mu }}_{c}^{⋆}-\mu_{0}^{⋆}}{1-\mu_{0}^{⋆}}.$$

**Figure 3:**
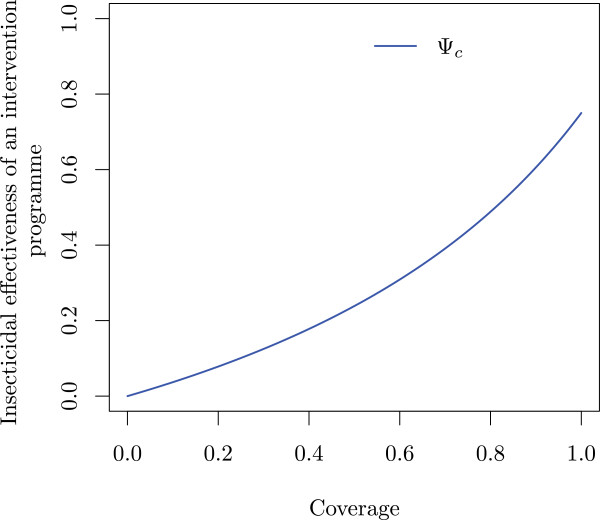
**Insecticidal effectiveness of intervention programmes depending on coverage.** The proportion deterred from entering, *δ*, is kept constant at 0.4, the proportion dead out of those that entered in an untreated control hut, *μ*_0_^⋆^, is kept constant at 0.20, and the proportion of mosquitoes dead in an experimental hut out of those that entered, *μ*_*t*_^⋆^, is kept constant at 0.8. Also, the proportion repelled (unfed alive mosquitoes) out of those that entered is kept constant at *ρ*_*t*_ = 0.3 and *ρ*_*0*_ = 0.1 for treated and control huts, respectively.

The relationship of Ψ_*c*_ with *c* is illustrated in Figure 3. With increasing *c*, the slope increases. When *c* is low, the slope is approximately $\frac{\mu_{t}^{⋆}-\mu_{0}^{⋆}}{1-\mu_{0}^{⋆}}/\frac{1-\rho_{0}}{\left(1-\delta \right)\left(1-\rho_{t}\right)}$; when *c* approaches 1, the slope is approximately $\frac{\mu_{t}^{⋆}-\mu_{0}^{⋆}}{1-\mu_{0}^{⋆}}\times \frac{1-\rho_{0}}{\left(1-\delta \right)\left(1-\rho_{t}\right)}$.

Quantity Ψ_*c*_'s validity as a measure for the insecticidal effectiveness of an intervention programme depends on the assumptions’ validity. On the one hand, insecticidal effectiveness of indoor intervention programmes targeting indoor biting mosquitoes may be lower if deterred mosquitoes shift to outdoor biting and resting. On the other hand, deterred mosquitoes are at higher risk of dying because of the increased search time, thus insecticidal effectiveness may be higher.

The quantity Ψ_*c*_ provides a ‘quick and dirty’ estimate of the potential insecticidal effectiveness of an intervention programme. The effectiveness of an intervention programme against disease transmission depends both on protection against bites and the insecticidal effectiveness. If detailed information about the vector population is available, such as data on (or good estimates of) daily survival rate (in the absence of intervention), anthropophily, endophagy, and endophily, more sophisticated models (e.g. [4,5]) can be used to predict impact on transmission.

### Protection against feeding in intervention programmes

Of those mosquitoes that enter a treated hut, the proportion of mosquitoes that feed is

(11) $$\phi_{t}=\frac{F_{t}}{E_{t}}=\phi_{t}^{⋆}\left(1-\rho_{t}\right),$$

where *F*_*t*_ is the number of fed females and *ϕ*_*t*_^⋆^ = *F*_*t*_/(*E*_*t*_ − *U*_*t*_) is the proportion of fed mosquitoes out of those that were not repelled after entry. Similarly, of those that enter the control hut, the proportion of mosquitoes that feed is estimated by

(12) $$\phi_{0}=\frac{F_{0}}{E_{0}}=\phi_{0}^{⋆}\left(1-\rho_{0}\right).$$

Following the same logic as for mortality (eq. 9), the mean feeding in an intervention programme is

(13) $${\bar{\phi }}_{c}^{⋆}=\frac{c\left(1-\delta \right)\left(1-\rho_{t}\right)\phi_{t}^{⋆}+\left(1-c\right)\left(1-\rho_{0}\right)\phi_{0}^{⋆}}{c\left(1-\delta \right)\left(1-\rho_{t}\right)+\left(1-c\right)\left(1-\rho_{0}\right)},$$

and the population level protection against feeding in an intervention programme is

(14) $${\text{Φ}}_{c}=\frac{\phi_{0}^{⋆}-{\bar{\phi }}_{c}^{⋆}}{\phi_{0}^{⋆}}.$$

The relationship of Φ_*c*_ with *c* is similar to that of Ψ_*c*_ with *c*. When *c* is low, the slope is approximately $\frac{\phi_{0}^{⋆}-\phi_{t}^{⋆}}{\phi_{0}^{⋆}}/\frac{1-\rho_{0}}{\left(1-\delta \right)\left(1-\rho_{t}\right)}$; when *c* approaches 1, the slope is approximately $\frac{\phi_{0}^{⋆}-\phi_{t}^{⋆}}{\phi_{0}^{⋆}}\times \frac{1-\rho_{0}}{\left(1-\delta \right)\left(1-\rho_{t}\right)}$. Note that Φ_*c*_ can be biased if a proportion of the mosquitoes that enter huts is exophagic.

### Endnotes

^a^Due to the possibility of mosquitoes escaping or being lost (e.g. through predation), depending on the hut design, the total number of mosquitoes caught in the experimental hut generally underestimates the number of mosquitoes that entered. The number escaping will be higher with increased excito-repellence, but lower with increased insecticidal effect.

^b^Repellence is defined here as the proportion of both unfed and live mosquitoes out of those that entered the hut and is not necessarily equal to the proportion of mosquitoes found in exit traps. All other mosquitoes collected (dead or both alive and fed) are presumed to have attacked the occupant of the hut.

^c^Attractants, with more mosquitoes entering treated huts than control huts, are not considered here.

^d^It is assumed here that mortality as measured in experimental huts is not biased upwards due to the experimental design. Mortality could be higher in exit traps than inside a control hut due to increased desiccation risk or prolonged exposure to insecticide, if exit traps are not emptied regularly [6].

## Competing interests

The authors declare that they have no competing interests.

## Authors’ contributions

All authors contributed equally. All authors read and approved the final version of the manuscript.
